# Integration of Computational Analysis and Spatial Transcriptomics in Single-cell Studies

**DOI:** 10.1016/j.gpb.2022.06.006

**Published:** 2022-07-25

**Authors:** Ran Wang, Guangdun Peng, Patrick P.L. Tam, Naihe Jing

**Affiliations:** 1 State Key Laboratory of Cell Biology, CAS Center for Excellence in Molecular Cell Science, Shanghai Institute of Biochemistry and Cell Biology, Chinese Academy of Sciences, University of Chinese Academy of Sciences, Shanghai 200031, China; 2 CAS Key Laboratory of Regenerative Biology, Guangdong Provincial Key Laboratory of Stem Cell and Regenerative Medicine, Guangzhou Institutes of Biomedicine and Health, Chinese Academy of Sciences, Guangzhou 510530, China; 3 Institute for Stem Cell and Regeneration, Chinese Academy of Sciences, Beijing 100101, China; 4 Embryology Research Unit, Children’s Medical Research Institute, University of Sydney, Sydney, NSW 2145, Australia; 5 School of Medical Sciences, Faculty of Medicine and Health, University of Sydney, Sydney, NSW 2145, Australia; 6 Guangzhou Laboratory, Guangzhou 510005, China

**Keywords:** scRNA-seq, Computational methodology, Spatial transcriptome, Data integration, Mathematical model

## Abstract

Recent advances of single-cell transcriptomics technologies and allied computational methodologies have revolutionized molecular cell biology. Meanwhile, pioneering explorations in spatial transcriptomics have opened up avenues to address fundamental biological questions in health and diseases. Here, we review the technical attributes of single-cell RNA sequencing and spatial transcriptomics, and the core concepts of computational data analysis. We further highlight the challenges in the application of **data integration** methodologies and the interpretation of the biological context of the findings.

## Introduction

High-throughput sequencing techniques, in particular single-cell omics analyses, have revolutionized molecular cell biology research. Single-cell RNA sequencing (scRNA-seq) is one of the most widely used single-cell analytical approaches. Since the first scRNA-seq profiling of only eight cells [1], the analysis has now been expanded to profile the transcriptome of around 2 million single cells [2]. The huge amounts of scRNA-seq data are of great potential to provide comprehensive cell atlas and cell connectivity for each biological system.

In order to make the most of these rich datasets, an effective computational analysis of single-cell data, including data quality control, read mapping, normalization, dimensionality reduction, clustering, heterogeneous cell population identification, differential gene expression, and trajectory inference, is an essential requisite to achieve a biologically meaning outcome of the study. To this end, a multitude of analytical tools are available, such as principal component analysis (PCA) [3], t-distributed stochastic neighbor embedding (t-SNE) [4], uniform manifold approximation and projection (UMAP) [5], and pseudotime inference [6]. Additionally, there are applications that integrate the analytical workflow for single-cell data, *e.g.*, Scran [7] and Seurat [8]. As each method has unique kernel algorithm and functionality, it is imperative to have a good understanding of computational attribute and biological implication of the methodology.

Further than identifying the signature features of major cell types in a cell population and characterizing the differentiation process, there is the aspiration to characterize the heterogeneity of cell types and the cell membership in a population [6]. The conventional single-cell assays necessitate the dissociation of tissue samples and does not register the tissue source and whereabouts of the single cells [9]. Since biological processes and cellular functions may be influenced by the immediate environment of cells, missing the spatial information during the isolation of cells is a major drawback of many single-cell studies [10]. Here entered the spatial transcriptome analysis that enables the elucidation of the cell type composition of a population in a defined tissue domain, organ, or body part of an organism. From these findings, inferences can made on cell states and lineage fate, and together with the additional temporal information, the activation of specific gene regulatory networks (GRNs) along a developmental trajectory in time and space of specific cell types, and emergence of cellular complexity in an organisms [8]. To accomplish this, analytical methods to achieve spatial resolution are developed, such as sequential fluorescence *in situ* hybridization (seqFISH) [11], multiplexed error-robust fluorescence *in situ* hybridization (MERFISH) [12], and slide sequencing (Slide-seq) [13]. Harnessing the power of high-resolution gene expression profiles and spatial reference systems by integrating the spatial coordinates gleaned from these studies with single-cell omics data would potentially empower the study of single-cell biology [14], [15].

This review focuses on bioinformatics methodology and spatial transcriptomics, which encompass most single-cell studies published to date. We outline the advances of single-cell analytical techniques, compare the technical attributes of various spatial omics technologies, and highlight the challenges and future trend of single-cell biology research.

## State-of-the-art analysis techniques

Recent advances in scRNA-seq technology provide unprecedented opportunities to investigate cell-to-cell heterogeneities, to identify new cell subtypes, and to model differentiation processes [16]. The analytical methodologies for scRNA-seq data differ dramatically from those for bulk RNA sequencing (RNA-seq). We summarize the core concepts of computational methodologies of scRNA-seq data analysis.

### Dimensionality reduction analysis for scRNA-seq data

**Figure 1:**
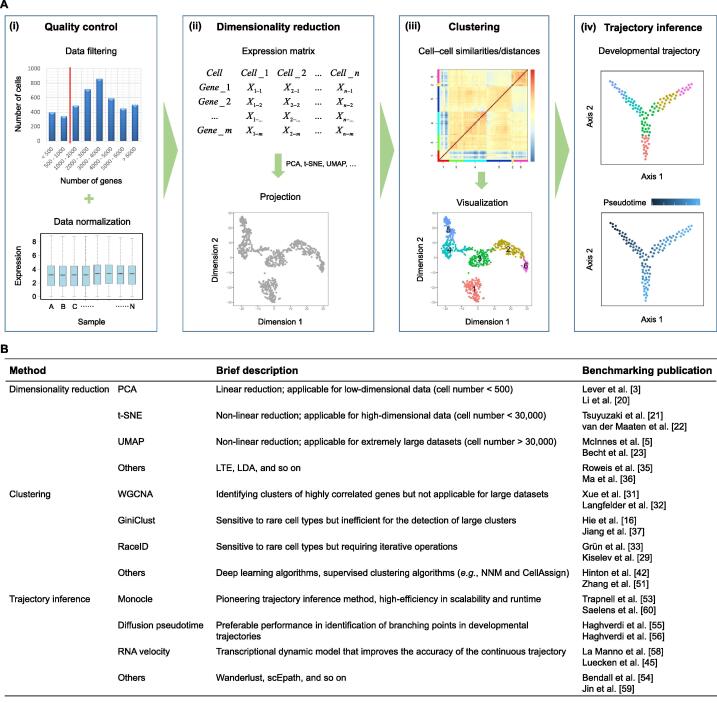
**State-of-the-art analysis techniques** **A.** Overview of the workflow for the computational analysis of scRNA-seq data. (i) Quality control; (ii) dimensionality reduction; (iii) cell–cell clustering; (iv) trajectory inference. **B.** Characterization of the state-of-the-art analysis techniques. PCA, principal component analysis; t-SNE, t-distributed stochastic neighbor embedding; UMAP, uniform manifold approximation and projection; WGCNA, weighted gene co-expression network analysis; RaceID, rare cell type identification; NNM, neural network model; LTE, locally linear embedding; LDA, linear discriminant analysis; scEpath, single-cell energy path.

In contrast to bulk RNA-seq, scRNA-seq generates complex datasets of high cell numbers and high-dimensional expression data [17], which require stringent quality control tests [18], [19] (Figure 1A, i). Dimensionality reduction is the primary step for scRNA-seq data analysis (Figure 1A, ii). PCA is one widely used dimensionality reduction method to extract variations and bring out significant patterns in a dataset [3]. PCA reduces the data dimension by geometrically projecting them onto fewer principal components (PCs), which act as summarizing the keynote features of data structure. Regarding the linear combination of the data’s primary variables, the top PCs, generally the top 1–3, reflect the foremost biological differences among cell clusters, since these PCs explain the major variances. Top PC loading genes, which can be interpreted as the signature genes of biological significance, account for the major variances for delineation of cell clustering, and can be used for further iterative clustering analysis [9], [20].

With the rapid advances of experimental techniques that could be applied to analyze an exponentially increasing number of cells per study, conventional methods may not be ideal for analyzing the complex multivariate data [21]. Among the computational tools and methodologies, the non-linear dimensionality reduction method [22], t-SNE, enables creating a single map that displays fine structure at many different levels (Figure 1A, ii). This is especially useful for high-dimensional data that comprise several distinct, but related, low-dimensional manifolds, such as graphics of objects from different classes seen from different viewpoints. For visualizing the structure of extremely large datasets, t-SNE uses random walk algorithm on neighborhood graphs to allow the potential and implicit structure of all of the data to be deduced from a concise sub-dataset.

Similar to t-SNE, UMAP is a latest non-linear dimensionality reduction method with great visualization capabilities [5]. UMAP is one of the fastest manifold learning implementations, and has a significant improvement over t-SNE in runtime and workload, especially for extremely large datasets [23], [24], [25], such as those in cell atlas studies (Figure 1A, ii). Of note, t-SNE and UMAP present similar arrangements of cell clusters, but display dissimilar morphological variations in visualization [25]. UMAP provides more specific and concrete visualization as well as preserving the global structures of the data. Additionally, UMAP’s branching clusters are able to highlight biological significances. In our own experience of single-cell data analysis, UMAP generates better results compared to t-SNE when the number of cells in scRNA-seq exceeds 30,000 (Figure 1B). To cope with high cellular throughput in recent single-cell studies, UMAP has taken the prior place of t-SNE to reduce dimensionality and to visualize the data structure [26], [27].

### Cell–cell clustering and cell type identification

Among the analytical methodologies of scRNA-seq data, clustering is equally important as dimensionality reduction. Within the lower dimensional space, most informative genes, *e.g.*, highly variable genes, were selected for further analysis [28]. Subsequently, clustering is the key step in categorizing cells and defining cell types (Figure 1A, iii), and this step requires thorough considerations of both biological and computational aspects. Many clustering methods are generic that can be applied to any dataset that possesses the measurement of distance/similarity between data nodes [29]. There are three major measurements for cell–cell distance: Euclidean distance, Pearson correlation coefficient (PCC), and Spearman’s rank correlation coefficient (SRCC) [30]. Euclidean distance represents the geometric similarities and amplifies the influence of highly variable genes, while the two latter measurements consider the scale invariance, in other words, they reflect the relative differences in values. For example, along with scRNA-seq technology, a popular clustering algorithm is weighted gene co-expression network analysis (WGCNA) [31], which generates weighted networks based on the PCC values and combines similar cells into larger clusters or divides heterogeneous clusters into smaller groups. Although designed for bulk RNA-seq analysis, this approach can be used for finding modules (clusters) of highly correlated genes in single cells or pseudocells generated by aggregating single-cell clusters, characterizing such modules using the module eigengene or GRNs, and identifying candidate biomarkers or therapeutic targets [32]. Grün et al. [33] sequenced the transcriptome of thousands of single cells isolated from mouse intestinal organoids, and by applying t-SNE analysis, they characterized major groups of cells. The unique element is that they incorporated an iterative algorithm to increase the detection sensitivity for rare cell populations. The optimized algorithm for rare cell type identification (RaceID) measures the Euclidean distance between single cells and enhances the sensitivity for detecting signature genes. In recent studies, RaceID has shown high accuracy in discovering rare and novel cell subtypes.

Single-cell analytical methodology is a fast-developing research field. Currently, a variety of integrative software packages for single-cell data analysis have been developed, with the most representative ones, including Scran [7], Seurat [8], and Scanpy [34]. These packages involve a step-by-step workflow for analyzing scRNA-seq data, aiming to identify cell–cell heterogeneities from transcriptomic measurements. Besides the computational approaches we summarized above, new approaches are emerging with unique kernel algorithms for data analyzing [16], [35], [36], [37] (Figure 1B). For example, single-cell regulatory network inference and clustering (SCENIC) [38] identifies co-expression modules, referring to as regulons, to construct the gene regulatory network [39], [40]. Census is a regression-based method to normalize scRNA-seq expression levels and detect gene regulatory changes [41]. Deep learning approaches have also been introduced into single-cell study, and neural network model (NNM) is a powerful mathematical algorithm that is able to capture and represent complex variable relationships [42], [43].

Although methodologies for single-cell analysis progress significantly, it is evident that the major challenge in analyzing scRNA-seq data is to understand the biological implication rather than computational outcome. The inference of cell states and functions, and the annotation of cell clusters require an in-depth understanding of the biological significance of each cluster [44]. In previous studies, researchers annotated cell types through unsupervised clustering with marker genes or projecting cells to existing cell atlases [24], [45], [46]. However, manual annotation is laborious, time-consuming, and error-prone due to lack of standard ontologies, especially for large datasets. To meet these challenges, supervised and semi-supervised clustering algorithms were developed [47]. In order to efficiently characterize cell types at the user end, SuperCT [48] and scPred [49] employ supervised training models to predict cell types. In comparison with unsupervised clustering methods, the supervised models showed notable performance in the prediction of cell subtypes. Representative semi-supervised clustering algorithms, Garnett [50] and CellAssign [51], are based on a paradigm of machine learning. Unlike unsupervised clustering, Garnett and CellAssign operate in an intuitive manner by incorporating prior knowledge of marker genes for interpretation and annotation of cell clusters. An additional advantage is that the analysis also expands the marker gene database for future application. With the increasing number of cell types being profiled, it would be useful to perform supervised and unsupervised clustering analyses simultaneously when analyzing new data to identify rare cell populations as well as transitional cell types during lineage differentiation.

### Developmental trajectory and pseudotime

In many biological systems, cells display a changing spectrum of states, which may mirror the differentiation process especially in development [52]. To infer the lineage history of single cells, it necessitates interrogating the continuity of cell states in scRNA-seq data. A unique advantage of scRNA-seq is that researchers can recapitulate developmental stages in a single experiment, given enough single cells and cell statuses sampled. Analytical algorithms underpinned by the concept of pseudotime have been applied to infer the developmental trajectory signposted by the transition of cell states during differentiation (Figure 1A, iv).

Pioneering trajectory inference methods such as Monocle [53] and Wanderlust [54] have been instrumental in the devising of pseudotime methodology to mimic the developmental process. Utilizing diffusion-like random walks to evaluate transitions across cell states, diffusion pseudotime (DPT) shows favorable performance in identification of branching points in developmental trajectories [55], [56]. RNA velocity employs a transcriptional dynamic model [57] that counts the unspliced and spliced mRNAs in scRNA-seq datasets [58] to enhance the fidelity of mapping the biological trajectory. Single-cell energy path (scEpath) calculates the entropy of dynamical process through statistical physics modeling, to predict the transition cell states and infer the lineage trajectories [59]. In essence, pseudotime displays an ordering of cell states by extracting the developmental information in the dataset, thereby allowing the identification of the cell types at early, intermediate, and end states of the pseudotime trajectory. From the cell orders, the gene regulatory networks that accompany the trajectory can be inferred [45]. While there are various methodologies to infer the lineage trajectory, no single method works best for all types of scRNA-seq data [6]. Different approaches have advantages and disadvantages in attributes, such as scalability, runtime, and resolution [60] (Figure 1B). Thus, the pseudotime methods for inferring cell trajectories should be employed iteratively with consideration of the alignment of biological context and computational output. Biological questions vary from one biological system to the others, and the choice of mathematical models is driven by the underlying assumptions for reconstructing lineage trajectories.

## Spatial transcriptomics

Cell type heterogeneity and cell fate acquisition are influenced by the environmental conditions and the interactive signals perceived by the cells at a specific location in the biological entity. However, current scRNA-seq technologies require tissue dissociation, resulting inevitably in the loss of spatial information on tissue architecture and organization. The methodology for collating the spatially resolved transcriptome involves bringing together the imaging technology and the profiling of gene expression, starting from classic low-plex RNA or protein expression assays to multimodalities.

### ***In situ*** spatial transcriptome analysis

**Figure 2:**
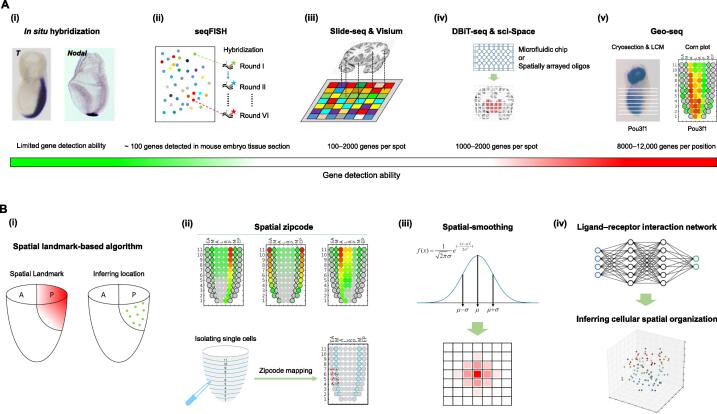
**The advances of spatial transcriptomics** **A.** Single-cell resolved spatial transcriptome technologies. In addition to the classic *in situ* hybridization (i), pioneering work includes seqFISH (ii), Slide-seq/Visium (iii), DBiT-seq/sci-Space (iv), and Geo-seq (v) greatly improve the spatial sensitivity and data depth. Whole-mount *in situ* hybridization images in (i) were obtained from the eMouseAtlas Project (www.emouseatlas.org). **B.** Combining scRNA-seq with spatial transcriptomics. (i) Inferring cellular localization based on spatial landmarks; (ii) zipcode mapping algorithm; (iii) spatial-smoothing algorithm; (iv) ligand–receptor interaction encoding cellular spatial organization. seqFISH, sequential fluorescence *in situ* hybridization; Slide-seq, slide sequencing; DBiT-seq, deterministic barcoding in tissue for spatial omics sequencing; Geo-seq, geographical positional sequencing; LCM, laser capture microdissection; A, anterior epiblast; P, posterior epiblast; L, left lateral epiblast; R, right lateral epiblast; M, mesoderm; EA, anterior endoderm; EP, posterior endoderm.

*In situ* hybridization is a road-tested technique that is used for localization and detection of specific DNA or RNA sequences in cells in segmented tissue domains, or in defined positions in the whole tissue [61], [62]. Leveraging the *in situ* patterns of spatially restricted genes as “landmarks”, individual cells with cell type-specific expression profiles identified by scRNA-seq analysis can be mapped to the inferred locations in the tissues [63] (Figure 2A, i), *e.g.*, the mapping of single cells in the brain of the marine annelid *Platynereis dumerilii* [64] (the spatial mapping will be discussed in more detail later).

The *in situ*-based reconstruction method is most effective where the tissue structure is stably stereotypic for the biological entity to assure universal consistency of position mapping among samples. These methods are, however, constrained by the scope of prior knowledge of cell markers, and only a customized set of markers can be assayed concurrently to establish high-confidence landmarks [9], which therefore greatly reduce the robustness of position mapping. To improve the detection throughput, recently developed methods of seqFISH [11] and MERFISH [12] use sequencing-by-hybridization techniques for transcriptomic quantification. For these techniques, cells fixed in space are subject to repeated hybridizations of fluorescently labeled DNA probes and rounds of imaging for registering the position of the *in situ* hybridization signals of up to hundreds of genes (Figure 2A, ii). While achieving an enhanced throughput compared to conventional *in situ* hybridization, the sensitivity of marker detection remains less than scRNA-seq.

Recently, the seqFISH technique is further enhanced by introducing pseudocolor and spectral overlapping [65], and this study reported the quantification of over 10,000 transcripts in individual cells. Multiplexed fluorescence *in situ* hybridization could image hundreds to thousands of genes simultaneously; however, this technology is constrained by the coverage of the marker gene sets, the availability of sensitive probes, and the requirement of high-resolution microscopy. Additionally, with increasing level of multiplexing, molecular markers starting to overlap and tight associations between cells may result in signal crowding, both leading to false-positive signals [66].

### Array-based spatial transcriptome analysis

To obtain a high-throughput, genome-wide profile of gene expression with spatial locations at cellular resolution, the latest spatial transcriptomic technology combines traditional histology technologies with massive throughput RNA-seq. For example, Slide-seq is a methodology which involves transferring mRNA from a tissue section onto a surface covered with DNA barcoded beads anchored at pre-defined positions, where the barcode registers the location of the captured mRNA [13] (Figure 2A, iii). Applying Slide-seq, cell types with characteristic gene expression profiles were identified at specific positions in the mouse cerebellum and hippocampus, thereby revealing the spatial pattern of gene expression of cells in the Purkinje layer and the temporal progression of cell type-specific responses to traumatic brain injury. Compared to the imaging-based methodologies, the density of sampling for Slide-seq analysis significantly improves the spatial resolution. However, the on-slide reaction is not uniformly efficient due to variations of cellular structures. Slide-seq detects 100–300 unique molecular identifiers (UMIs) per cell, which are equivalent to ∼ 100 genes [67]. The impact of sparsity of data signals over noise is yet unsolved, and the number of genes that can be simultaneously assayed is still limited. Slide-seq V2 has improved library generation and bead synthesis to increase RNA capture efficiency [68]. While the gene detection ability has been enhanced (5–10-fold of Slide-seq), the low-throughput has confounded the efficiency of cell type clustering.

Originated from a similar platform as Slide-seq, 10X Genomics Visium Spatial Gene Expression Solution (Visium) captures the transcriptome from cells at their positions in an intact tissue section to generate a spatial display of the gene activity (Figure 2A, iii). Using the Visium technology, the spatial topography of gene expression of the human dorsolateral prefrontal cortex (DLPFC) has identified laminae-enriched expression signatures of the six-layered DLPFC, and refined the molecular connectivity to previous laminar markers [69]. Compared with Slide-seq, Visium has an improved throughput and enables gene expression analysis at the transcriptome level on intact sections of tissues. Nevertheless, this technology is still discommoded by unsatisfactory gene detection ability (500–2000 genes per spatial spot) and incompatibility to all tissue types, meanwhile, the limited spatial resolution also makes it insufficient to elucidate cell-to-cell heterogeneity [70].

In recent spatial transcriptomics studies, a number of outstanding methodologies and improvements have been developed that enable high-throughput *in situ* profiling of gene expression. Additional array-based platforms, such as high-definition spatial transcriptomics (HDST) [71] and spatial enhanced resolution omics-sequencing (Stereo-seq) [72] offer high-resolution and high-density coverage, by refining the spatial resolution to 2 μm subcellular range, though the limited gene detection ability related to the sequencing depth remains unresolved.

### Direct spatial barcoding captures tissue coordinates

Pioneering attempts toward spatial transcriptome analysis were based on either multiplexed fluorescence *in situ* hybridization or barcoded bead array [73], [74]. These methodologies are still technically demanding, requiring complicated processing steps and hard pressed by the low mRNA capture efficiency. A nascent technology takes the approach of deterministic barcoding in tissue for spatial omics sequencing (DBiT-seq) [75], instead of transferring mRNAs onto a solid-phase substrate. DBiT-seq places microfluidic chips with perpendicular channels against a fixed tissue section to directly barcode the biomolecules (Figure 2A, iv). In this regard, DBiT-seq obviates the need for sophisticated processes of sequential hybridization or bead decoding by directly capturing the approximate coordinates of the cells upon sequencing. Conceptually similar to DBiT-seq, sci-Space method [76] employs the spatially arrayed unique combination of oligonucleotides (oligos) that are transferred to nuclei of cells by diffusion. The nuclei are then extracted from the tissue section and prepared for scRNA-seq (Figure 2A, iv). The spatial coordinates of large-scale regions can be retrieved by sci-Space, which meets a need of extended coverage that cannot be fulfilled by other methodologies [13].

Both DBiT-seq and sci-Space have strengths and limitations. DBiT-seq greatly improves spatial resolution close to single-cell level, but does not resolve single cells directly. High-resolution immunofluorescence on the same tissue section simultaneously can facilitate cell segmentation. sci-Space focuses on high-throughput screens and can cover large tissue domains, but the spatial resolution (∼ 200 μm) is limiting. Decreasing spot size and increasing spot density may refine resolution, but the gene detection ability would be compromised. The capturing rates (1000–2000 genes per spot) of both technologies would benefit from further optimization.

### Laser capture microdissection records geographical location

Spatial locations of cells in tissues strongly influence cellular functions. A key prerequisite for single cell research is the efficient harvesting of single cells of interest from a tissue or cell group. Laser capture microdissection (LCM) enables capture of target cells with structural and spatial information retained [77], [78], but the resolution remains relatively low. The limitation lies in the number of cells captured (ranging from hundreds to thousands for library preparation) and the coverage of profiling limiting to a smaller gene set, making it difficult to discern the heterogeneity and cell-to-cell interactions within a sampled population.

Geographical positional sequencing (Geo-seq), a technique combining scRNA-seq and LCM, was developed [14]. This technique features optimized preservation tissue morphology and enhanced RNA integrity to enable low input RNA-seq analysis, making it possible to analyze the transcriptome of limited materials of as few as ten cells sampled from defined locations in the tissue. Using Smart-seq2 for full-length scRNA-seq [79], the gene detection efficiency has significantly increased to detect up to 8000–12,000 genes in each LCM sample. The depth of sequencing data enables the elucidation of gene structure based on splicing information. Incorporating the spatial information enables the construction of the 3D spatial and quantitative transcriptome atlas [15] (Figure 2A, v). Through bioinformatics analysis, Geo-seq data can be mined to infer the gene regulatory networks that underpin the molecular architectures of embryonic development from preimplantation to gastrulation in mice [39].

Geo-seq is potentially applicable to investigate a variety of biological questions, such as cell fate determination, cell type heterogeneity, and gene regulatory network. By applying Geo-seq, a spatial transcriptome map of early post-implantation mouse embryos utilizing data collated from sections along their proximal–distal axis has revealed that podocalyxin exocytosis is associated with the polarization of extra-embryonic tissue and the formation of pro-amniotic cavity [80]. Geo-seq analysis of the caudal hematopoietic tissue (CHT) of zebrafish has unveiled the spatio-temporal dynamics of the expansion of hematopoietic stem cell and progenitor cell in CHT and the accompanying gene regulatory networks, which points to a strong association between hematopoietic stem cell differentiation and cell cycle status [81].

### Combining scRNA-seq with spatial transcriptomics

Other than the “direct” *in situ* single-cell analytical techniques (seqFISH, MERFISH, DBiT-seq, *etc*.), leveraging the attributes of scRNA-seq and spatial transcriptomics to retrieve from the integrated datasets the spatial positions of single cells offers another avenue for single-cell spatial transcriptomics study [9]. For example, a core feature of Seurat package is inferring cell location by integrating scRNA-seq data with the spatial pattern of *in situ* RNA expression [63]. Seurat first divides the embryo or tissue structure into high-resolution bins followed by binarization of the expression of *in situ* hybridization signals of specific landmark genes. As a result, the landmarks are normalized as “on” or “off” within each spatial bin and the reference positioning system is thereby constructed. By aligning the expression of landmark genes of single cells to the reference gene set, the locations of single cells can be determined (Figure 2B, i). However, the limited number of spatial landmark genes and the different platforms for generating the references and the single-cell data may impact the precision of spatial mapping. In the latest version of Seurat, through identification of pairwise correspondences between single cells, termed “anchors”, across diverse datasets, a shared space that improves the precision of inferring single-cell locations, can be constructed [8]. Similarly, the distributed mapping (DistMap) scores [82], by binning the entire *Drosophila* embryo into refined expression regions and computing the Matthews correlation coefficients (MCCs) between the expression values of spatial landmarks for every cell–region combination, can predict multiple likely positions for each cell, instead of assigning to a single location. The higher the resolution of the reference positioning system, the higher is the precision of inferred localization. Employing high content seqFISH data as a reference system increases the number of spatial-specific markers compared to conventional *in situ* hybridization. For instance, by integrating scRNA-seq data of “Gastrulation Atlas” [24] and quantified seqFISH data, a spatially resolved map of gene expression at quasi single-cell resolution can be constructed for the mouse embryo [83].

One drawback of the landmark-based algorithms is the limited availability of known landmarks. To mitigate this shortcoming, Geo-seq incorporates a spatial signature gene (zipcode) mapping protocol that computationally allocate single cells to spatial positions (Figure 2B, ii). The unbiased identification of “zipcodes” embedded in Geo-seq data enhances the imputation of the positions of single cells. Over 90% of the single cells isolated from known positions can be mapped correctly with high degrees of precision to the original position in the embryo [15]. By applying zipcode mapping protocol, stem cells in culture or cells from germ layers of a gastrulating embryo can be mapped to their best-fit positions in the embryo [84], [85]. By incorporating a spatial-smoothing algorithm, the mapping efficiency and spatial confidence intervals can be further enhanced. Gaussian distribution-based spatial simulation that mirrors the spatial dispersity of single-cell mapping (Figure 2B, iii) showed that the simulation of diffused domain has a significantly improved fidelity compared with other spatial mapping algorithms (Wang and Jing, unpublished). The methodologies for combining scRNA-seq data and spatial transcriptomics are still in its infancy. Efficient extraction of both spatial coordinates and spatial signature/zipcode genes continues to be a challenging task for computational biology [86]. Mathematical modeling may also be introduced to gain a deeper level of understanding of the spatial information.

### Mathematical models for spatial organization

Besides experimentally recording the spatial information of individual cells, computational biologists have attempted to reconstruct spatial map of single cells mathematically. The core idea is to use a reference atlas of signature genes as an “anchor” to assign spatial coordinates to each cell [63], [64], [82]. However, such methodologies rely heavily on the coverage of spatial marker genes, even with a specific combination of reference genes which may not be adequate to tag every spatial position. To resolve the cell positions, a *de novo* spatial reconstruction (novoSpaRc) algorithm was applied to *de novo* construct the cartography of single-cell gene expression, without any reliance on prior knowledge [87]. The assumption for novoSpaRc is that neighboring cells display more similar transcriptional identities than cells farther apart and the physical distance between cells increases with their biological distance in gene expression space. Applying novoSpaRc on published single-cell datasets, cells of mammalian intestinal epithelia [88] and liver lobules [89] can be re-positioned. Consistent with the notion of structural correspondence, classified cell types showed distinct pairwise distances.

Similar to the spatial–distance approach of novoSpaRc, the cellular spatial organization mapper (CSOmap) algorithm assumes that ligand–receptor interaction information encodes the cellular spatial organization [90], for inferring *de novo* cellular interactions from scRNA-seq data. CSOmap is built on the hypothesis that ligand–receptor interactions mediate cellular self-assembly rather than the transcriptome profiles (Figure 2B, iv). CSOmap suggests that transcriptome-similar cells often, but not always, have similar spatial locations [91], [92]. Decoding intercellular communication networks in the perspective of ligand–receptor interactions have important biological implication [93]. Recently, a growing number of methodologies that model how gene expression of a cell is influenced by interacting cells are developed. Based on cellular communication models, methods such as CellPhoneDB, NicheNet, SingleCellSignalR, and iTALK [94], [95], [96], [97] can predict ligand–receptor links between interacting cells by integrating their expression profiles with prior knowledge on gene regulatory networks and signaling activities. Although these methodologies did not implement the analysis of the space-resolved reorganization of single cells, they open new opportunities to dissect the complexity, heterogeneity, and molecular dynamics of cell–cell communication. Further development of the spatial algorithms of cellular communication models is anticipated in future studies.

Mathematical modeling provides important insights into spatial transcriptomics, but the pseudo-space may not depict the complexity of biological contexts. For example, different cell types may require higher-order regulatory networks or exhibit distinct principles of spatial organization. Incorporating biological insights into mathematical models, and in combination with algorithms of machine learning [98], will be a priority of methodology development.

## Outlook and challenges

Single-cell analytical techniques that enable high-throughput profiling of multi-omics have revolutionized molecular cell biology. There is a compelling need to efficiently analyze the large-scale single-cell omics data for thousands to millions of cells. On one hand, the ability to unravel biological complexity is empowered by the accessibility to big data. On the other hand, higher computing power is needed to raise the ability to cluster cell populations comprehensively and identify rare cell types [29]. Visualizing and deciphering the results continues to be testing. Classic linear methods, such as hierarchical clustering and PCA, are insufficient to capture relationships between cells in large datasets accurately due to the high levels of data variation. Deep learning and non-linear techniques are more flexible (t-SNE, UMAP, *etc*.), as they can identify all variations, extract significant features, and visualize the structure of very large datasets aesthetically.

With the advances of single-cell analytical methodologies, another challenge is the choosing of suitable analytic methodology for the single-cell data. The most commonly used methods, such as Scran [7], Seurat [8], and Scanpy [34], each with the unique kernel algorithm may have an impact on the outcome. As these analytical methods offer many parameters that can be customized to define the criteria of normalization and resolution of clustering, the selection of parameters could have an unintended effect on the outcome [99]. Thus, it is important to inform computational analysis and data interpretation with biological guidance, particularly in the clustering and definition of cell types.

Time-series scRNA-seq experiments provide voluminous information on the gene sets that are dynamically activated, and their role in cellular interaction and development. Given the complexity of dynamic biological processes, a discrete clustering or classification of cells may not fully mirror the full spectrum of cell states. Pseudotime study of time-series data will be critical in future studies. Current pseudotime methods, such as Monocle [53] and DPT [55], simulate a biological process such as cell differentiation by ordering cells along a model of developmental trajectory [100]. This allows the identification of intermediate cell states and the branching points of the trajectory, besides inferring the beginning and end points. Innovation in pseudotime methodology, for revealing time-series activity of transcription factors (TFs) [58] and the gene regulatory networks anchored by these TFs, as well as the modulation of signaling activity, is crucial for understanding the dynamics of the molecular function [101].

A pressing question in biological research is “what defines a cell type and cellular functionality?” [102]. Previous single-cell analyses often try to define a cell type using marker genes or transcriptome identity [103], [104]. However, cell fate decision and cellular heterogeneity can be influenced by the environmental factors and the signals in the neighborhood of the cell [39]. Most of the single-cell studies lack the spatial information of cells. To answer the question “what is a cell type?”, knowledge of the environmental input to guide cell differentiation and lineage development is a vital prerequisite in single-cell research [105]. How to computationally retrieve the original spatial coordinates of individual cells requires an integrated approach of spatial transcriptome and precise gene detection at single-cell resolution [39]. Once generated, the spatially resolved single-cell maps will facilitate the computational inference of gene regulation networks and signaling networks. To address the question of “cellular functionality”, single-cell multi-omics data beyond the transcriptome have to be collated and be integrated with the spatio-temporal information into a biomolecular cell atlas that would inform the functional output of the cells in development and homeostasis. Additionally, when the mathematical models can be complemented by experimental validation, single-cell technologies would elevate the dimensionality of our fundamental understanding of cell and developmental biology [10].

## CRediT authorship contribution statement

**Ran Wang:** Conceptualization, Investigation, Writing – original draft. **Guangdun Peng:** Writing – review & editing. **Patrick P.L. Tam:** Writing – review & editing. **Naihe Jing:** Supervision, Writing – review & editing. All authors have read and approved the final manuscript.

## Competing interests

The authors have declared no competing interests.
